# Synergistic effect of tartaric acid with 2,6-diaminopyridine on the corrosion inhibition of mild steel in 0.5 M HCl

**DOI:** 10.1038/srep33305

**Published:** 2016-09-15

**Authors:** Yujie Qiang, Lei Guo, Shengtao Zhang, Wenpo Li, Shanshan Yu, Jianhong Tan

**Affiliations:** 1 School of Chemistry and Chemical Engineering, Chongqing University, Chongqing 400044, China; 2 School of chemical engineering, Sichuan University of Science and Engineering, Zigong 643000, China; 3 National-municipal Joint Engineering Laboratory for Chemical Process Intensification and Reaction, Chongqing 400044, China; 4 School of Materials and Chemical Engineering, Tongren University, Tongren 554300, China; 5 School of Chemistry and Chemical Engineering, Yangtze Normal University, Chongqing 408100, China

## Abstract

The inhibitive ability of 2,6-diaminopyridine, tartaric acid and their synergistic effect towards mild steel corrosion in 0.5 M HCl solution was evaluated at various concentrations using potentiodynamic polarization measurements, electrochemical impedance spectroscopy (EIS), and weight loss experiments. Corresponding surfaces of mild steel were examined by atomic force microscope (AFM), field emission scanning electron microscope (FE-SEM), energy dispersive X-ray spectroscopy (EDX), X-ray photoelectron spectroscopy (XPS) analysis. The experimental results are in good agreement and reveal a favorable synergistic effect of 2,6-diaminopyridine with tartaric acid, which could protect mild steel from corrosion effectively. Besides, quantum chemical calculations and Monte Carlo simulation were used to clarify the inhibition mechanism of the synergistic effect.

Due to its excellent mechanical property and low price, mild steel is widely used as the construction and engineering material in a variety of chemical and petrochemical industries^1^,^2^,^3^. However, the major disadvantage of mild steel is its limited resistance to corrosion under harsh environments. Consequently, the use of hydrochloric acid in acid cleaning, descaling, pickling, and oil well acidizing, causes severe corrosion attack on mild steel^4^. Up to now, the addition of organic inhibitors is one of the most efficient methods for preventing steel from corrosion^5^,^6^,^7^,^8^. Hence, investigating corrosion inhibitors of mild steel in aggressive acid media are important not only in practical applications but also for academic value.

The inhibitive ability of organic compounds for metal corrosion is usually attributed to their adsorption ability on metal surfaces, which can block the active sites on metal surfaces and thereby suppress the corrosion attack. Generally, the adsorption of organic molecule on metal surface depends mainly on the surface charge of metal, the chemical structure of organic molecule and the type of aggression medium^9^,^10^,^11^. It is well known that organic compounds containing polar functional groups, several heteroatoms (i.e. sulfur, nitrogen, oxygen) and conjugated double bonds, generally exhibit excellent inhibition efficiency^12^,^13^,^14^,^15^. Therefore, many organics have been explored as corrosion inhibitors in the last few decades. However, the usage of a majority of these inhibitors has been restricted due to the high price and toxicity^13^. Growing environmental concern have promoted researchers to focus on the investigation of eco-friendly corrosion inhibitors and their synergistic effects^16^,^17^,^18^,^19^,^20^.

2,6-Diaminopyridine (a common organic dye)^21^, tartaric acid (a common beverage additive)^22^ are both cheap, low cost, environment-friendly organics. But their poor inhibition efficiency is not enough to protect corrosion of mild steel. Therefore, the purpose of the present work is to survey the inhibitive ability of 2,6-diaminopyridine, tartaric acid and their synergistic effect towards mild steel corrosion in 0.5 M HCl solution, which has not been reported previously. Potentiodynamic polarization, electrochemical impedance spectroscopy (EIS), weight loss, EDX, AFM, FE-SEM techniques were employed to evaluate the inhibition performance. In addition, quantum chemical calculations and Monte Carlo simulation^23^,^24^,^25^ were further adopted to add theoretical support for experimental results and investigate the mechanism of the synergetic effect.

## Experimental

### Materials and sample preparation

The mild steel coupons having a composition (wt.%) of 0.20% C, 0.17% Si, 0.12% Mn, 0.05% P, 0.02% S, and balance Fe were mechanically cut into 1.00 cm^3^ dimensions for the electrochemical experiments. The exposed surface area of electrochemical specimen was 1 cm^2^, while the remainder was embedded by epoxy. Besides, the dimension of steel specimens for weight loss experiments were 3.00 cm × 1.50 cm × 1.50 cm. Prior to each experiment, the specimens were abraded consecutively with emery papers from 400 to 2000 grit, then washed with distilled water, degreased with acetone, finally dried at room temperature.

**Figure 1:**
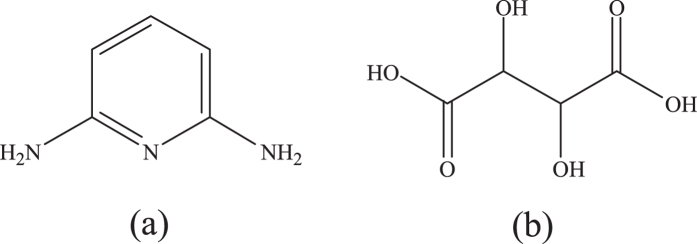
Chemical structures of the investigated inhibitors, (**a**) DAP, (**b**) TTA.

The corrosive medium 0.5 M HCl was prepared by analytical grade hydrochloric acid. 2,6-diaminopyridine (DAP, Aladdin, 98%) and tartaric acid (TTA, Aladdin, 99.5%) shown in Fig. 1 were used as received. The testing solution was prepared using 0.5 M HCl solution with different concentrations (DAP: 1, 2, 4, 10 mM, TTA: 0.5, 1, 2, 5 mM) of the inhibitors and combination of them (Num−1: 1 mM DAP + 0.5 mM TTA, Num−2: 2 mM DAP + 1 mM TTA, Num−3: 4 mM DAP + 2 mM TTA, Num−4: 10 mM DAP + 5 mM TTA). The solution without addition of inhibitors was deemed as blank for comparison. All experiments were performed at 298 ± 1 K via thermostat water bath.

### Weight loss measurements

Cleaned and weighed mild steel samples in triplicate were immersed in 0.5 M HCl solution with and without different concentrations of DAP, TTA and combination of them for 8 h at 298 K, respectively. Then the samples were taken out, scrubbed with a bristle brush, cleaned by distilled water and acetone, then dried and weighed by analytical balance.

### Electrochemical tests

Electrochemical measurements were carried out in a traditional three-electrode cell by CHI660B electrochemical workstation. Mild steel coupon was used as a working electrode. Saturated calomel electrode (SCE) and Pt electrode were treated as reference and counter electrodes, respectively. All potentials in the present study were measured with respect to SCE.

Prior to each measurement, the working electrode was immersed in the aggressive media for 30 min until a steady state of open circuit potential (OCP) was obtained. Subsequently, EIS measurements were carried out at the OCP over a frequency range of 100 kHz to 0.01 Hz, with a sinusoidal AC perturbation of 10 mV peak-to-peak. The impedance data were fitted using Zsimpwin 3.10 software. Finally, polarization curves were recorded at a scan rate of 2 mV s^−1^ in the potential range of ± 250 mV versus the OCP. In order to guarantee a favorable reproducibility, the same experiment was carried out for 3 times.

### Surface characterization

The surface morphology of the steel specimens before and after immersed in 0.5 M HCl solution without and with 10 mM DAP, 5 mM TTA and combination of them for 8 h at 298 K were characterized by field emission scanning electron microscope (FE-SEM, JEOL−JSM−7800F, Japan) at high vacuum and atomic force microscopy (AFM, Seiko-SPIN-3800N, Japan) using tapping mode, respectively.

The chemical composition of synergistic adsorbed film of DAP and TTA on mild steel was detected by XPS (VSW Spectrometer) employing Al Ka (1486.6 eV) as the incident radiation source and the binding energy of C 1 s (285.0 eV) was used as an internal reference. The relevant sample of mild steel was prepared via immersion in 0.5 M HCl with 10 mM DAP and 5 mM TTA meanwhile. After removal from the test solution, steel sample was rinsed with distilled water and dried under vacuum for XPS measurement.

### Calculation methods

Quantum chemical calculations were conducted with DMol^3^ module in Materials Studio software 7.0. Geometrical optimizations and frequency calculations were accomplished by the generalized gradient approximation (GGA) functional of Becke exchange plus Lee−Yang−Parr correlation (BLYP) method within the density functional theory (DFT). Fine convergence accuracy and global orbital cutoffs were employed. To get more reliable data, the solvent effect was considered by using conductor-like screening model (COSMO) and defining water as the solvent. In addition, the quantum chemical parameters calculated from the optimized structures were analyzed.

The adsorption behavior of DAP and TTA molecules on iron surface was investigated using Monte Carlo simulations via adsorption locator module from Accelrys Inc. The simulations were performed in a simulation box (19.8 × 19.8 × 40.1 Å) with periodic boundary conditions to simulate a representative part of an interface devoid of any arbitrary boundary effects. COMPASS (Condensed-phase Optimized Molecular Potentials for Atomistic Simulation Studies), a first ab initio forcefield that could accurately predict chemical and condensed-phase properties for plenty of chemical systems, was employed to optimize the structures of all components of the system.

## Results and Discussion

### Weight loss measurements

The inhibition effect of DAP, TTA and synergistic effect of them at various concentrations towards corrosion of mild steel in 0.5 M HCl was investigated by weight loss methods at 298 K after 8 h immersion. The relevant corrosion rate *v* (mg m^–2^ h^–1^), inhibition efficiency (*η*) and synergistic coefficient (*S*) at different concentrations are calculated as follows^26^ and listed in Table 1.


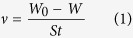



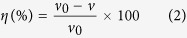



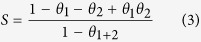


where *S* in equation (1) is the total surface area of the samples, *W*_0_ and *W* are the average weight of samples before and after exposure to 0.5 M HCl solution, respectively, *t* is the immersion time, *v*_0_ and *v* are the corrosion rates of mild steel samples without and with inhibitor, respectively, *θ*_1_, *θ*_2_, and *θ*_1+2_ are the surface coverages (defined by *η* in weight loss measurements) of DAP, TTA and coexistence of them.

As seen from Table 1 that corrosion rates decrease while the inhibition efficiencies increase with incremental concentration of both inhibitors. However, inhibition efficiencies of single inhibitor are rather low, indicating weak inhibitive ability of these organics at all concentrations. Interestingly, the inhibitive efficiencies of combination of both compounds are higher than that of two inhibitors alone in corresponding concentration, suggesting a better inhibitive capacity towards corrosion. It is discovered that the maximum efficiency reaches 92.1% with synergistic effect, while single DAP and TTA is 74.5% and 68.8%, respectively. Indeed, the values of *S* are higher than 1 at most concentrations (1 mM, 2 mM and 10 mM DAP), indicating that the interaction between DAP and TTA is a synergistic effect. However, when the concentration of DAP reaches 4 mM, the value of S becomes less than 1. This means that an antagonistic effect occurs between DAP and TTA^27^.

### Potentiodynamic polarization curves

**Figure 2:**
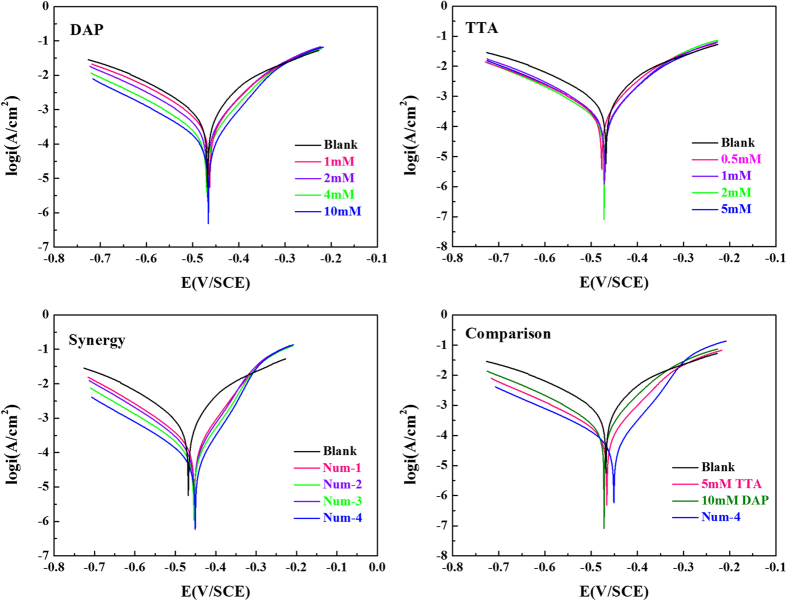
Potentiodynamic polarization curves recorded for mild steel electrode in 0.5 M HCl solution containing different concentrations of (**a**) DAP, (**b**) TTA, (**c**) Synergy and (**d**) Comparison at 298 K.

Tafel polarization curves recorded on mild steel electrode in 0.5 M HCl solution without and with different concentrations of DAP, TTA and DAP + TTA are shown in Fig. 2a–c. Figure 2d also displays their optimum curves for comparison. The key electrochemical parameters, including corrosion potential (*E*_corr_), corrosion current density (*i*_corr_), anodic and cathodic Tafel slope (*β*_a_, *β*_c_), and inhibition efficiency (*η*) derived from these figures are given in Table 2. The values of *η* are calculated as follows^28^,^29^:


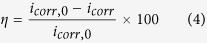


where *i*_corr,0_ and *i*_corr_ represent uninhibited and inhibited current densities of the mild steel specimen, respectively.

In Fig. 2a,b, with increase in the concentration of these compounds, the polarization curves move weakly to lower current densities, which indicates that corrosion rate of mild steel has been suppressed to a certain extent. For both DAP and TTA, the corrosion potentials are almost same as that of the blank solution, while cathodic and anodic current densities of the polarization curves are both reduced^30^,^31^. The results reveal that these organics acts as mixed-type corrosion inhibitors by inhibiting the hydrogen reduction and also mild steel anodic dissolution^32^. But it is clear that the protection is not enough for severe steel corrosion in 0.5 M HCl solution. With regard to DAP and TTA alone, the polarization curves of combination of both inhibitors move remarkably to low current densities (Fig. 2c). Besides, as seen in Fig. 2d, superior polarization curves in synergy were obtained than inhibitors present alone. The results indicate that DAP and TTA display favorable synergistic effect to prevent mild steel from corrosion due to the adsorption and the formation of protective film on the electrode surface^33^,^34^.

Each value of *β*_c_ in Table 1 is exactly similar with the others, which indicates that the mechanism of the hydrogen evolution reaction is not changed by the addition of these inhibitors. The hydrogen evolution was probably reduced by the surface blocking effect. Table 1 reveals that corrosion current densities (*i*_corr_) decrease delicately with single inhibitor. In the contrast, the value of *i*_corr_ decrease obviously in the presence of both organic inhibitors meanwhile. These values of *i*_corr_ continue to decrease and *η* that calculated from *i*_corr_ increase with increasing concentration of these inhibitors. The maximum values of *η* are 82.0% with DAP, 71.3% with TTA and 92.5% with synergistic effect. It can be concluded that more effectively barrier film of inhibitor molecules are formed on the specimen surface by synergy, thus blocks the active sites on the steel surface to protect mild steel from corrosion^35^,^36^.

### Electrochemical impedance spectroscopy

**Figure 3:**
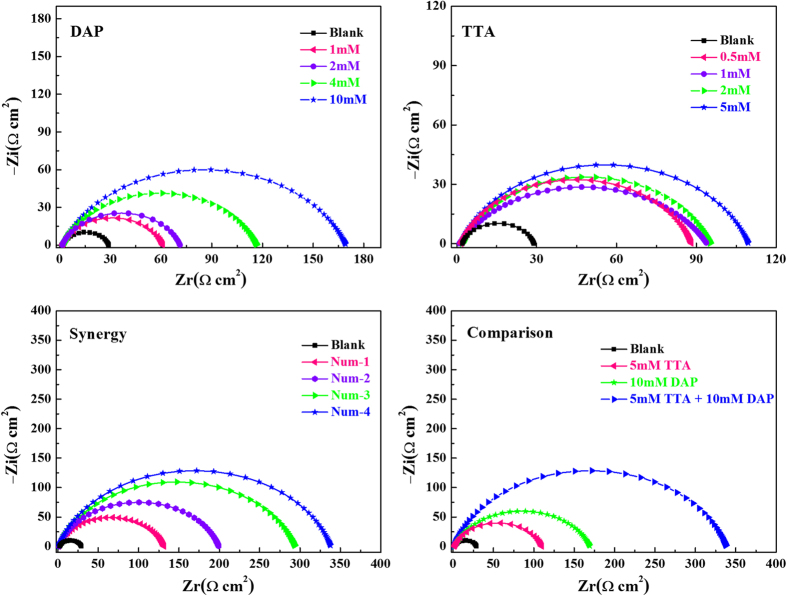
Nyqusit plots for mild steel in 0.5 M HCl solution without and with different concentrations of (**a**) DAP, (**b**) TTA, (**c**) Synergy and (**d**) Comparison at 298 K.

Electrochemical impedance spectroscopic investigations of mild steel in 0.5 M HCl solution in the absence and presence of different concentrations of inhibitors were performed to verify the results of weight loss and polarization experiments^37^,^38^,^39^. The related Nyquist plots are given in Fig. 3.

**Figure 4:**
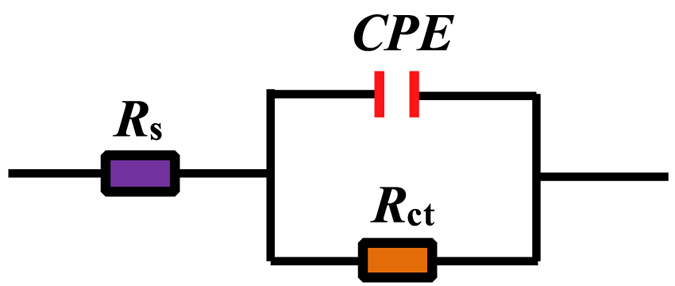
Equivalent circuit used to fit the EIS data.

As shown in Fig. 3, all Nyquist plots exhibit one single capacitive loop, indicating that the corrosion of mild steel in 0.5 M HCl with and without protection is mostly controlled by charge transfer process and double layer capacitance^40^. In addition, these impedance spectra show a similar shape at all tested concentrations, which suggests that corrosion mechanism is quite not changed^19^,^41^,^42^. Compared with the blank solution, the diameter of the semicircles increases slightly with the addition of single DAP or TTA. However, the diameter of curves increases dramatically with combination of both inhibitors, showing a better inhibitive capacity with synergistic effect^14^. Besides, these diagrams are depressed semicircles with the centers under the real axis, which may be attributed to the frequency dispersion effect^27^. This phenomenon is due to the surface roughness, the surface chemical heterogeneity, grain boundaries, and adsorption–desorption process of the organic molecules on metal surface^43^,^44^. Accordingly, a constant phase element (*CPE*) must be introduced to the equivalent circuit as shown in Fig. 4 to accurately fit the impedance data. Here, *R*_s_ is the solution resistance and *R*_ct_ represents the charge transfer resistance.

The impedance of *CPE* is defined as follows^11^,^43^,


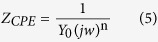


where *Y*_0_ is the modulus of the *CPE*, *w* is the angular frequency, *j* is imaginary number (*j*^2^ = −1), *n* is the deviation parameter in regard to a phase shift. When *n* = −1, the *CPE* represents an inductor, for *n* = 0, a pure resistor, and for *n *= 1, a pure capacitor.

The obtained data are given in Table 3. The values of *C*_dl_ are calculated as follows^7^,


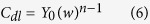


where *w*_max_ = 2π*f*_max_ and *f*_max_ is the frequency at the maximum value of the imaginary component of the impedance spectrum. The values of *η* of these inhibitors and synergy for the mild steel electrode in 0.5 M HCl solution are calculated from the following equation^45^,


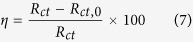


where *R*_ct_ and *R*_ct,0_ are the charge transfer resistances for mild steel in 0.5 M HCl media with and without inhibitors, respectively.

As can be seen from Table 3, it is clear that the *R*_ct_ values increase with increasing concentration of inhibitors. Especially, the increasing trend of *R*_ct_ values for combination of DAP and TTA is more obvious. These results may be attribute to the adsorption of the inhibitors onto the metal/solution interface. The adsorption behavior is more remarkable in the presence of two inhibitors meanwhile, indicating that a strong protective film could be formed by the synergistic adsorption of these inhibitors on mild steel surface. In addition, the values of *C*_dl_, which can be explained by the Helmholtz model as equation (7)^10^, exhibits a decreasing trend with increase in concentration of inhibitors.


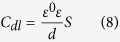


where *d* is the thickness of electric double-layer, *S* is the surface area of mild steel electrode exposed to 0.5 M HCl solution, *ε*^0^ and *ε* show the permittivity of the air and the local dielectric constant, respectively. Therefore, the decrease of *C*_dl_ values can attribute to the adsorption of these inhibitor molecules, which reduces the exposed electrode surface area in aggressive solution and thus retards steel corrosion effectively^42^,^46^. Furthermore, water molecules on the surface of mild steel are replaced gradually by inhibitor molecules, leading to lower local dielectric constant and thicker electric double-layer. All these factors cause the decease of *C*_dl_^47^,^48^.

Consequently, inhibition efficiencies increase with increasing the concentration of inhibitors and reach 83.9% for DAP at 10 mM, 75.0% for TTA at 5 mM and 92.0% for synergy at 10 mM DAP + 5 mM TTA, respectively. It indicates that synergistic effect display effective protection towards corrosion of mild steel in 0.5 M HCl medium. These results of the EIS studies are in perfect accordance with the results obtained from the weight loss and polarization measurements.

### FE-SEM and EDX analysis

**Figure 5:**
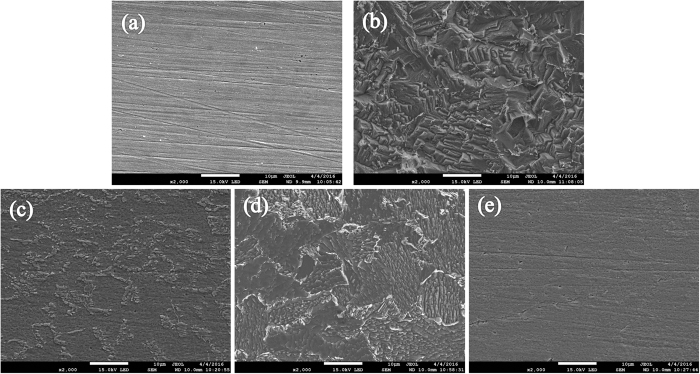
FE-SEM images of (**a**) freshly polished mild steel specimen and the specimens immersed in 0.5 M HCl solution (**b**) without and with (**c**) 10 mM DAP, (**d**) 5 mM TTA and (**e**) 10 mM DAP + 5 mM TTA for 8 h at 298 K.

Figure 5 shows the high-definition micrographs of the mild steel specimens before and after immersion in 0.5 M HCl solution for 8 hours in absence and presence of inhibitors at optimum concentration. The steel surface in 0.5 M HCl solution without inhibitors (Fig. 5b) becomes scratched, rough and porous obviously. With addition of single 10 mM DAP or 5 mM TTA, the surfaces of the specimens shown in Fig. 4c,d are protected but still corroded in some extent. However, the specimen surface with synergistic effect (Fig. 5e) is smoother than counterpart with one inhibitor alone, and the micro-image is nearly the same as the polished mild steel (Fig. 5a). So it can be concluded that corrosive attack is considerably reduced by combination of DAP and TTA, indicating high-efficiency protective film on the mild steel surface are formed by these organic molecules.

**Figure 6:**
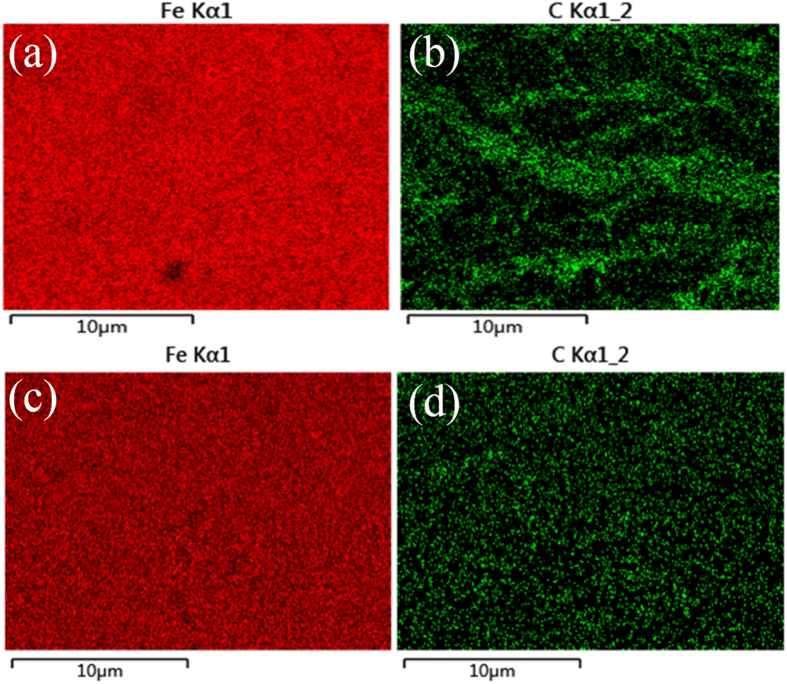
EDX elemental mapping of Fe and C for the uninhibited (**a**,**b**) and inhibited (**c**,**d**) mild steel surface by synergistic effect.

Figure 6 shows the elemental mapping images of Fe and C for mild steel surface without and with protection of synergistic effect. C element is not distributed evenly in the unprotected mild steel surface after immersed in 0.5 M HCl solution. But Fe and C elements are both homogeneously distributed in the inhibited steel surface, which suggests corrosion are reduced effectively by synergy.

### Atomic force microscopy study

**Figure 7:**
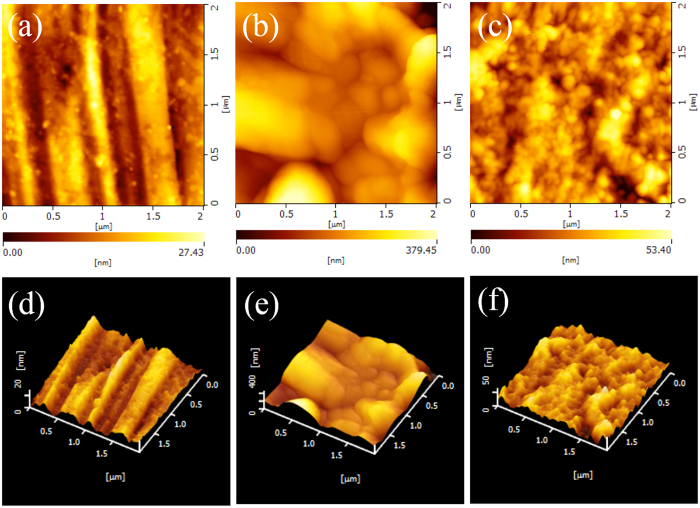
Two-dimensional and three-dimensional AFM images of: (**a**,**d**) polished mild steel, and (**b**,**e**) unprotected and (**c**,**f**) protected mild steel by synergistic effect in 0.5 M HCl solution at 298 K.

AFM is considered to be a powerful tool to explore the surface morphology at nano- to microscale and has become a new choice to discuss the corrosion process at the metal/solution interface^49^,^50^,^51^. The two-dimensional (2D) and three-dimensional (3D) AFM graphs of mild steel surface before and after immersed in 0.5 M HCl solution without and with protection of synergistic effect at 298 K are shown in Fig. 7(a–e), respectively. It can be seen that the AFM pattern of the freshly polished steel surface looks mostly uniform with only some tiny scratches, while the surface of sample exposed to 0.5 M HCl solution shows a rather rough and porous structure with large and deep pores due to the aggressive attack by corrosive media. However, with addition of both inhibitors meanwhile, the surface becomes relatively flat and smoother, which clearly reveals that the corrosion rate of steel sample significantly decreases (Fig. 7c,f).

The mean roughness of the mild steel surface before 0.5 M HCl treatment, after unprotected treatment and protected treatment are 4.11 nm, 17.22 nm and 6.69 nm, respectively. The maximum peak-to-valley height (P–V) of synergistic surface are also reduced to 64.77 nm from 160.7 nm of uninhibited surface. These parameters suggest that the surface appears smoother and hence corrosion rate decreases due to the protective film formation by adsorption of inhibitor molecules on the surface of mild steel^35^. This finding together with the results obtained from FE-SEM and electrochemical tests suggest that an ordered and dense layer could be formed by combination of two inhibitors so that mild steel is protected availably.

### XPS analysis

**Figure 8:**
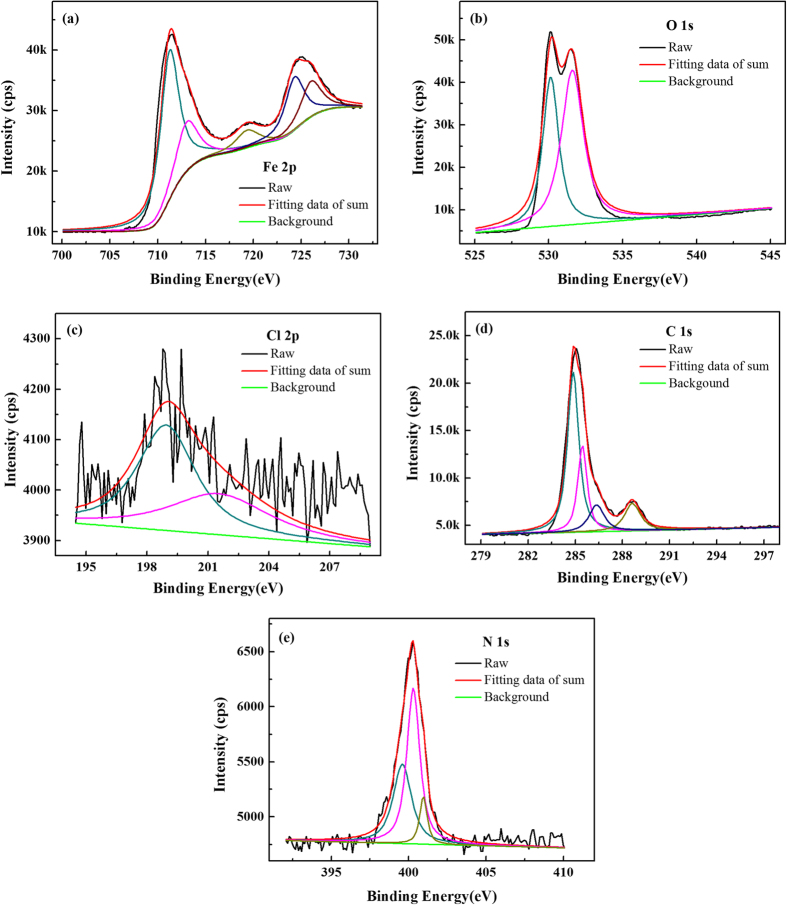
High-resolution X-ray photoelectron deconvoluted profiles of (**a**) Fe 2p, (**b**) O 1s, (**c**) Cl 2p, (**d**) C 1s, and (**e**) N 1s for mild steel in 0.5 M HCl with synergistic protection.

X-ray photoelectron spectroscopy (XPS) analysis was performed to get insight into the chemical nature of the inhibitors/mild-steel interface and to investigate the adsorption mechanism of the synergistic effect. The obtained high-resolution XPS spectra (Fe 2p, O 1s, Cl 2p, C 1s, N 1s) of mild steel surface in 0.5 M HCl in the presence of both DAP and TTA meanwhile are illustrated in Fig. 8. These spectra show complex forms which have been assigned to the corresponding species through a deconvolution fitting procedure.

The Fe 2p spectrum as shown in Fig. 8a presents mainly five peaks. The high peak at lower binding energy (711.2 eV) corresponds to metallic iron^52^. The peak located at 713.0 eV is attributable to Fe 2p3/2, and the small peak at 719.40 eV corresponds to the satellite of Fe^3+^ 53. Besides, the peaks at 724.3 eV and 726.0 eV can be ascribed to Fe 2p1/2 due to the presence of iron in the form of Fe_3_O_4_, α-Fe_2_O_3_ and FeOOH^54^. The O 1s spectrum (Fig. 8b) is deconvoluted into two peaks at around 530.1 eV and 531.6 eV. The first peak (530.1 eV) is related to ferric oxides such as Fe_2_O_3_ and/or Fe_3_O_4_, while the second peak at 531.6 eV is assigned to OH^−^ of hydrous iron oxides (i.e., FeOOH)^55^. The Cl 2p shown in Fig. 8c is best fitted into two components located at around 198.9 eV for Cl 2p3/2 and 201.5 eV for Cl 2p1/2^56^. As seen in Fig. 8d, the peaks at 284.9 eV for aromatic rings and 285.4 eV for C = N^+^ prove that the adsorption of DAP molecules on steel surface^57^. Indeed, the peak at 288.6 eV associated with C = O indicates that TTA molecules have adsorbed on mild steel surface^58^. The N 1s spectra may be fitted into three main peaks at 399.6 eV, 400.3 eV and 400.9 eV corresponding to C−N−metal connection, coordinated nitrogen atom and the = N^+^− in the pyridine ring^59^. The presence of the nitrogen species bonded in different forms with the mild steel surface demonstrates that these inhibitors can get adsorbed both physically as well as chemically^60^.

### Quantum chemical calculations

**Figure 9:**
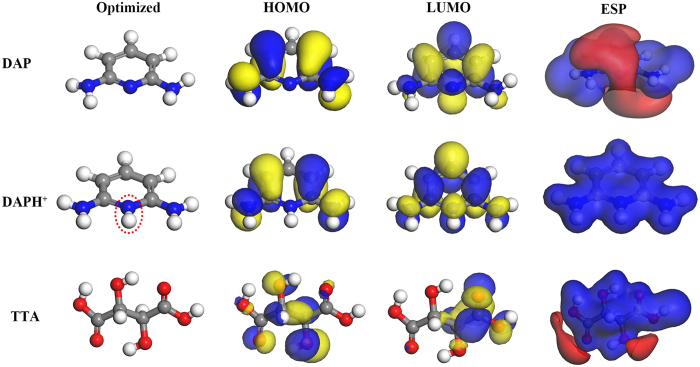
Optimized molecular structure, HOMO orbital, LUMO orbital and electrostatic potential (ESP) map of DAP, DAPH^+^ and TTA molecule.

Quantum chemical calculations have been used to investigate the relationship between molecular/electronic structure of an organic inhibitor and its inhibition efficiency. Figure 9 shows optimized structures, frontier molecular orbital density distribution and electrostatic potential (ESP) map of TTA molecule, the neutral and protonated forms of DAP molecule. Indeed, the obtained quantum chemical indices, such as the energy of the highest occupied molecular orbital (*E*_HOMO_), the energy of the lowest unoccupied molecular orbital (*E*_LUMO_), energy gap (Δ*E* = *E*_LUMO_ − *E*_HOMO_) and dipole moment (*μ*) for the DAP, DAPH^+^ and TTA molecule are listed in Table 4.

As seen in Fig. 9, both HOMO and LUMO are localized evenly on the entire DAP molecule, suggesting a parallel adsorption of DAP molecules onto mild steel surface, which is similar as the protonated form of DAP (DAPH^+^). For TTA, HOMO spread mainly on the hydroxyl groups while the distribution of LUMO is about half of the TTA molecule. Thus it is reasonable to assume that DAP and DAP^+^ contain more adsorption centers, leading to higher adsorption ability than TTA^29^. In addition, more dark red (negative) regions associated with nucleophilic reactivity in the ESP map of DAP indicate that DAP molecules are readily transfer or sharing free electron pairs to mild steel (electrophilic agent) to form covalent bonds compared with TTA^61^. Especially the whole region of the pronated form of DAP molecule is blue (positive) region with electrophilic reactivity.

As known that low value of *E*_LUMO_ indicates the ability of the molecule as an electrons-accepter, whereas high value of *E*_HOMO_ means a strong electron-donating ability to the suitable acceptor^3^,^30^. Reasonably, the order of *E*_HOMO_ (DAP > TTA) in Table 4 is accordance with inhibition efficiencies obtained above. Interestingly, the values of both *E*_LUMO_ and *E*_HOMO_ for DAPH^+^ are decreased owing to be pronated. However, a low Δ*E* value for DAPH^+^ still be observed and close to DAP from Table 4, showing that these species can absorb easily on metal surface than TTA^35^,^62^. Furthermore, the dipole moment is also an important index and a low value of dipole moment favors the accumulation of organic molecules on the metal surface thereby increasing the inhibition ability^63^. The values of *μ* for natural and pronated form of DAP are lower than that for TTA, which indicates the order of inhibition efficiency obtained is theoretically confirmed.

### Monte Carlo simulation

**Figure 10:**
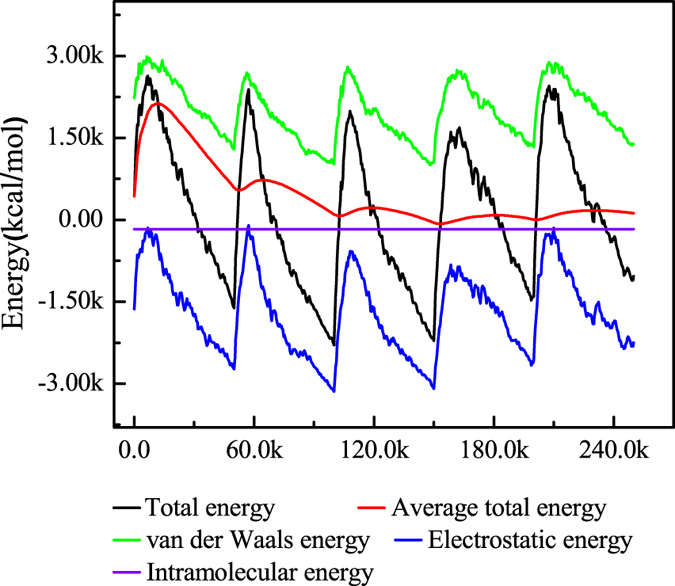
Total energy distribution for Inhibitor/H_2_O/Fe(110) system during energy optimization process (*x*:*y*:*m*:*n* = 2:0:1:400).

Figure 10 shows total energy distribution for Inhibitor/H_2_O/Fe(110) system during energy optimization process using *x*:*y*:*m:n* = 2:0:1:400 (as defined in Table 5) model as an example. The outputs and descriptors obtained from the Monte Carlo simulation, such as total energy, adsorption energy, rigid adsorption energy and deformation energy are given in Table 5.

Total energy, which defined as the sum of adsorption energy and the internal energy of the sorbate, gave a high absolute value in two inhibitor coexistence meanwhile than only single inhibitor, which demonstrates a more stable adsorption configuration^64^. In such a condition, the substrate energy (steel surface) is deemed as zero. Furthermore, dE_ad_/d_Ni_ is the differential adsorption energy, indicating the energy of removing a adsorbate of a particular component. The low absolute value of dE_ad_/d_Ni_ for H_2_O in Table 5 means that water molecule can be replaced gradually by inhibitor molecules. Adsorption energy, composed of two parts: the energy of adsorbing the sorbate onto the steel surface in its input conformation (rigid adsorption energy), and a small deformation energy owing to relaxation of the sorbate on Fe(110) surface, is the most important parameter of adsorption. High absolute value of the adsorption energy reflects a strong adsorption behavior^63^. Therefore, synergistic effect of studied compounds exhibits greater inhibition abilities as compared to single DAP or TTA. Besides, the order of adsorption energy is accordance with inhibition efficiency obtained from experimental results.

**Figure 11:**
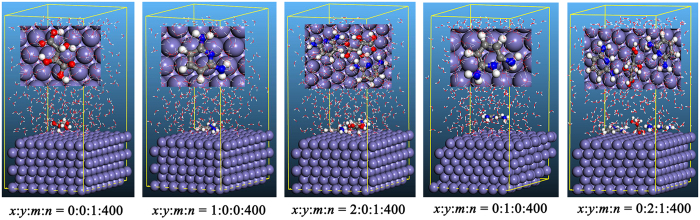
Top and Side views of the most stable configurations for the adsorption of inhibitors on Fe(110) interface obtained using Monte Carlo simulations.

Figure 11 shows top and side view of the most stable adsorption configurations for the studied inhibitors onto Fe(110) surface with five situation. It can be seen that studied molecules adsorbed on Fe(110) surface with a parallel mode in all circumstances. Especially, a synergistic adsorption of DAP and TTA molecule occurred when combination of both inhibitors, indicating a favorable inhibition ability for steel corrosion is reasonable.

### Mechanism of corrosion inhibition

Generally, the mechanism of corrosion inhibition in acid medium is clarified by the adsorption of organic compound onto the metal surface. The inhibition efficiency of the inhibitor is related to many factors including metal type, corrosive medium, molecular size, number of adsorption centers, the electronic structure, and chemical properties of the inhibitor, mode of interactions between inhibitor and metal surface^65^. According to the results obtained from the experiments and theoretical studies, synergistic effect of DAP and TTA exhibits favorable inhibition ability, whereas single inhibitor with dissatisfied inhibition efficiency. This may be due to the coadsorption of DAP and TTA molecules, which is either competitive or cooperative^66^. In cooperative adsorption, the electron-rich species are absorbed on the steel surface and the low electron negative or neutral species are adsorbed subsequently. In competitive adsorption, different inhibitor molecules may be adsorbed at different sites on the surface of mild steel^67^. Hence, both cooperative and competitive adsorption may occur simultaneously. However, as can be seen in Fig. 11, the competitive adsorption between the DAP molecules and TTA molecules dominates over the cooperative adsorption.

## Conclusions

DAP and TTA as mixed-type inhibitors inhibit both anodic and cathodic processes on the corrosion of mild steel in 0.5 M HCl solution, and their inhibitive efficiencies increase with incremental concentration. Synergistic effect of TTA and DAP exhibits a better inhibitive ability than single inhibitor. Besides, morphology analysis together with the results obtained from electrochemical tests suggest that an ordered and dense layer could be formed by synergistic effect so that mild steel is protected availably. Furthermore, XPS analysis and theoretical studies indicate that the synergistic protection is mainly dominated by competitive adsorption between the DAP molecules and TTA molecules physically and chemically.

## Additional Information

**How to cite this article**: Qiang, Y. *et al*. Synergistic effect of tartaric acid with 2,6-diaminopyridine on the corrosion inhibition of mild steel in 0.5 M HCl. *Sci. Rep.* **6**, 33305; doi: 10.1038/srep33305 (2016).

## Figures and Tables

**Table 1:** Corrosion parameters obtained from weight loss measurements for mild steel in 0.5 M HCl solution without and with different concentrations of DAP, TTA and synergistic effect for 8 h at 298 K.

*C* (mM)	DAP		*C* (mM)	TTA		*C* (mM)	Synergy		*S*
	*v* (mg m^−2^ h^−1^)	*η* (%)		*v* (mg m^−2^ h^−1^)	*η* (%)		*v* (mg m^−2^ h^−1^)	*η* (%)	
Blank	273.5	/	Blank	273.5	/	Blank	273.5	/	/
1 mM	152.6	44.2	0.5 mM	128.0	53.2	Num-1	68.1	75.1	1.049
2 mM	114.6	58.1	1 mM	100.9	63.1	Num-2	40.2	85.3	1.052
4 mM	85.3	68.8	2 mM	89.7	67.2	Num-3	32.3	88.2	0.867
10 mM	69.7	74.5	5 mM	85.3	68.8	Num-4	21.6	92.1	1.007

**Table 2:** Potentiodynamic polarization parameters for mild steel in 0.5 M HCl solution without and with different concentrations of DAP, TTA and combination of them at 298 K.

*C* (mM)	*E*_corr_ (mV/SCE)	*i*_corr_ (mA cm^−2^)	SD^a^	*β*_c_ (mV s^−1^)	*β*_a_ (mV s^−1^)	*η* (%)
Blank	−468	0.595	0.011	−125.3	79.9	/
DAP						
1	−463	0.381	0.008	−122.4	82.6	36.0
2	−468	0.301	0.007	−122.7	79.4	49.4
4	−469	0.172	0.005	−119.8	71.7	71.1
10	−466	0.107	0.007	−128.7	63.7	82.0
TTA						
0.5	−477	0.259	0.009	−123.3	68.2	56.5
1	−471	0.219	0.006	−111.5	72.0	63.2
2	−491	0.198	0.008	−113.5	67.6	66.7
5	−472	0.171	0.004	−115.3	63.4	71.3
Synergy						
Num-1	−453	0.147	0.006	−107.1	63.4	75.3
Num-2	−454	0.093	0.002	−85.3	55.7	84.3
Num-3	−454	0.074	0.004	−95.8	57.5	87.6
Num-4	−451	0.045	0.004	−104.5	54.2	92.5

^a^SD, standard deviation.

**Table 3:** Impedance parameters of mild steel in 0.5 M HCl solution in the presence and absence of DAP, TTA and combination of them at 298 K.

*C* (mM)	*R*_s_ (Ω cm^2^)	*R*_ct_ (Ω cm^2^)	SD	*Y*_0 _× 10^−6^ (S s^n^ cm^−2^)	n	*C*_dl_ (μF cm^−2^)	*η* (%)
Blank	2.03	26.97	0.31	267.3	0.83	98.2	/
DAP							
1	1.71	59.30	0.36	256.0	0.80	96.6	54.5
2	1.58	69.66	0.25	237.2	0.81	94.8	61.3
4	1.80	114.8	0.48	233.2	0.80	91.3	76.5
10	1.62	167.9	0.41	216.5	0.79	90.3	83.9
TTA							
0.5	1.60	86.65	0.39	246.8	0.82	98.1	68.9
1	0.91	93.06	0.63	403.6	0.70	94.2	71.0
2	1.97	93.27	0.61	266.6	0.80	94.7	71.1
5	1.42	108.2	0.97	223.0	0.81	91.4	75.0
Synergy							
Num-1	0.83	130.5	1.14	210.8	0.83	90.9	79.3
Num-2	0.92	198.5	1.67	165.0	0.82	80.1	86.4
Num-3	1.02	292.7	1.46	157.4	0.82	78.3	90.8
Num-4	0.82	337.4	1.52	128.8	0.83	68.0	92.0

**Table 4:** Quantum chemical parameters for the DAP, DAPH^+^ and TTA molecule.

	*E*_HOMO_ (eV)	*E*_LUMO_ (eV)	Δ*E* (eV)	*μ* (Debye)
DAP	−4.49	−0.79	3.70	0.28
DAPH^+^	−9.38	−6.19	3.19	3.10
TTA	−6.66	−2.03	4.63	3.35

**Table 5:** Outputs and descriptors calculated by the Mont Carlo simulation for adsorption of inhibitors on Fe(110) (in kcal/mol).

DAP:DAPH^+^: TTA:H_2_O^b^	Total energy	Adsorption energy	Rigid adsorption energy	Deformation energy	H_2_O: d*E*_ad_/d*N*_i_	DAPH^+^: d*E*_ad_/d*N*_i_	DAP: d*E*_ad_/d*N*_i_	TTA: d*E*_ad_/d*N*_i_
0:0:1:400	−5190.3	−5208.1	−5519.2	311	−16.4	/	/	−105.7
1:0:0:400	−5307.4	−5212.4	−5519.3	306	−15.1	/	−64.4	/
2:0:1:400	−5465.9	−5293.6	−5610.7	317	−15.1	/	−76.9	−102.4
0:1:0:400	−5274.1	−5223.4	−5519.2	296	−14.4	−107.9	/	/
0:2:1:400	−5393.0	−5279.3	−5512.2	233	−13.1	−81.5	/	−92.9

^b^DAP:DAPH^+^:TTA:H_2_O = *x*:*y*:*m:n* means the adsorbates contain *x* DAP, y DAPH^+^, *m* TTA, and *n* water molecules.
